# *Listeria monocytogenes* Isolated from Illegally Imported Food Products into the European Union Harbor Different Virulence Factor Variants

**DOI:** 10.3390/genes9090428

**Published:** 2018-08-23

**Authors:** Kathrin Rychli, Beatrix Stessl, Kati Szakmary-Brändle, Anja Strauß, Martin Wagner, Dagmar Schoder

**Affiliations:** Institute of Milk Hygiene, University of Veterinary Medicine Vienna, Veterinärplatz 1, 1210 Vienna, Austria; kathrin.rychli@vetmeduni.ac.at (K.R.); beatrix.stessl@vetmeduni.ac.at (B.S.); kati.szakmary-braendle@vetmeduni.ac.at (K.S.-B.); anja.strauss@lva.at (A.S.); martin.wagner@vetmeduni.ac.at (M.W.)

**Keywords:** *Listeria monocytogenes*, genotyping, virulence factors, internalin A, listeriolysin O, ActA, food, illegal, neglected routes, European Union

## Abstract

Unregulated international flow of foods poses a danger to human health, as it may be contaminated with pathogens. Recent studies have investigated neglected routes of pathogen transmission and reported the occurrence of *Listeria monocytogenes* in food illegally imported into the European Union (EU), either confiscated at four international airports or sold illegally on the Romanian black market. In this study we investigated the genotype diversity and the amino acid sequence variability of three main virulence factors of 57 *L. monocytogenes* isolates. These isolates were derived from 1474 food samples illegally imported into the EU and originated from 17 different countries. Multilocus sequence typing revealed 16 different sequence types (STs) indicating moderate genotype diversity. The most prevalent STs were ST2, ST9, and ST121. The pulsed-field gel electrophoresis (PFGE) analysis resulted in 34 unique pulsotypes. PFGE types assigned to the most prevalent STs (ST2, ST9, and ST121) were highly related in their genetic fingerprint. Internalin A (InlA) was present in 20 variants, including six truncated InlA variants, all harbored by isolates of ST9 and ST121. We detected eight ST-specific listeriolysin O (LLO) variants, and among them, one truncated form. The actin-assembly-inducing protein ActA was present in 15 different ST-specific variants, including four ActA variants with an internal truncation. In conclusion, this study shows that *L. monocytogenes*, isolated from illegally imported food, have moderate genotype diversity, but diverse virulence factors variants, mainly of InlA.

## 1. Introduction

Zoonooses are infectious diseases of animals that can be transmitted to humans via animal contact or by consuming contaminated water or food of animal origin. While microorganisms do not respect national borders, susceptibility to zoonotic infections is dependent upon local factors, such as hygiene practices, population or herd immunity, and the presence or absence of transmission vectors. Consequently, unregulated international flow of foods via breaches to land, sea and air route restrictions has the potential to endanger human and animal health [1].

Indeed, there have already been several disease outbreaks in humans within the European Union (EU) due to illegally imported foods of animal origin [2,3,4].

International airports serve as important bottlenecks for the illegal importation of products of animal origin. The urge to smuggle foreign foods may reflect the appeal of European migrants and travelers for exotic items for either nostalgic or religious reasons [5], or merely the desires of tourists for novel souvenirs. However, lack of food refrigeration during transit most likely facilitates pathogen enrichment beyond levels that would otherwise be anticipated.

In order to meet the challenges of protecting human and animal health, and at the same time, to ensure the continued free movement of goods and people, the EU has introduced and financed the research project PROMISE (protection of consumers by microbial risk mitigation through combating segregation of expertise). A main objective of this project was to assess the microbial risk associated with the uncontrolled import of foodstuffs, especially products of animal origin, to the customs union. Significantly, once illegal imports enter the EU they may circulate unimpeded over 28 member states and the three European Free Trade Association (EFTA) countries of Liechtenstein, Norway, and Switzerland.

One such exemplary foodborne pathogen that has raised significant attention is *Listeria monocytogenes*. *L. monocytogenes* is a facultative, intracellular pathogen responsible for listeriosis, a disease associated with severe localized and generalized infections in most domestic animals and humans [6,7]. Susceptible consumers of contaminated foods are typically infants, pregnant women, and the elderly, debilitated, and immunocompromised [8]. Once infection is established, the invasive form of listeriosis in humans is associated with a fatality rate of between 15–30% [9]. The three main clinical manifestations of human listeriosis are maternofetal listeriosis, blood stream infection, and meningoencephalitis [8]. However, unlike most other bacteria, *L. monocytogenes* is able to survive and grow at low temperatures, high salt concentrations, and low pH values; conditions widely adopted to safeguard food-keeping qualities [10,11].

Previously published studies reported the occurrence of this major pathogen in food confiscated at EU airports from air passenger luggage originating from non-EU countries. Despite a range of existing regulations, recent spot-checks of the luggage of 61,355 passengers from 240 flights from non-EU countries at Vienna International Airport, involving 600 food samples, revealed the presence of foodborne bacteria in 5% of all samples [12]. The highest prevalence of foodborne pathogens in this study was attributed to *L. monocytogenes* at 2.5%. Similarly, related studies identified *L. monocytogenes* in 10% of foods seized at the International Airport of Bilbao, Spain [13] and in 1.4% of illegally imported food at two German airports (Frankfurt International and Berlin-Schönefeld Airport) [5]. Additionally *L. monocytogenes* was detected in 7.5% of foods illegally sold at the Romania–Republic of Moldova border [14].

The aim of this study was to investigate the genotype diversity and virulence gene variations of 57 *L. monocytogenes* isolates from 1474 food samples illegally imported into or sold within the EU. Therefore we performed multilocus sequence typing (MLST), pulsed-field gel electrophoresis (PFGE), and analyzed the full-length amino acid sequences of three essential virulence genes: InlA, required for the invasion of *L. monocytogenes* into the host cells [15]; listeriolysin O (LLO), a pore-forming toxin of the cholesterol-dependent cytolysin (CDC) family, essential for phagosomal escape, intracellular growth, and cell-to-cell spread [16]; and the actin assembly-inducing protein ActA. ActA is responsible for bacterial movement and cell-to-cell spread [17], and has recently been shown to be also involved in biofilm formation and aggregation as well as long-term colonization of the gastrointestinal tract [18].

## 2. Materials and Methods

### 2.1. Listeria monocytogenes Isolates

All *L. monocytogenes* isolates used in this study were isolated within the Seventh Framework Programme (FP7) EU project “*PROMISE—protection of consumers by microbial risk mitigation through segregation of expertise*” from food products illegally imported into the EU. Briefly, food samples were confiscated by EU border inspection forces from passengers’ luggage travelling through four international airports: Vienna International Airport (Austria) [12], Bilbao Airport (Spain) [13], Frankfurt International Airport, and Berlin-Schönefeld Airport (both Germany) [5]. Additionally, food products were sampled from a black market in Galati, Romania [14,19]. These food samples were analyzed for the presence of *L. monocytogenes* according the ISO 11290-1 (1996).

### 2.2. Multilocus Sequence Typing and Pulsed-Field Gel Electrophoresis Typing

Multilocus sequence typing (MLST), which is based on seven housekeeping genes (*abcZ* (ABC transporter), *bglA* (beta glucosidase), *cat* (catalase), *dapE* (succinyl-diaminopimelate desuccinylase), *dat* (d-amino acid aminotransferase), *ldh* (l-lactate dehydrogenase), *lhkA* (histidine kinase)) was performed according to Ragon et al. [20]. PCR products were sequenced (LGC Genomics, Berlin, Germany), and an allele number was assigned to each housekeeping gene. Sequence type (ST) numbers were attributed to each distinctive allele combination using the Institute Pasteur *L. monocytogenes* MLST database. A detailed protocol of the MLST procedure, including primers, PCR conditions, and allelic type, are available at the Institute Pasteur website (http://bigsdb.pasteur.fr/listeria/listeria.html). Classification of the STs into clonal complexes (CCs) was performed according to Canticelli et al. [21]. MLST data of *L. monocytogenes* isolates from food confiscated at the airport Vienna, Austria [12]; Bilbao, Spain [13]; and the black market in Galati, Romania [19]; were included. The MLST genes were concatenated for each strain, resulting in 3288 nucleic acids and aligned using MUSCLE implemented in MEGA7 using the Tamura–Nei evolutionary model and the maximum likelihood treeing method [22]. Gapless alignments were checked manually.

Pulsed-field gel electrophoresis (PFGE) typing applying the restriction enzymes *Asc*I and *Apa*I was performed according to the standardized PulseNet International protocol [23]. 

Restricted DNA was electrophoresed on 1% (w/v) SeaKem gold agarose (Lonza Group AG, Basel, Switzerland) in 0.5× TBE at 6 V/cm on a Chef DR III system (Bio-Rad Laboratories, Inc., Hercules, CA, USA). A linear ramping factor with pulse times from 4.0 to 40.0 s at 14 °C, and an included angle of 120° was applied for 22.5 h. Gels were stained with ethidium bromide (Sigma Aldrich, St. Louis, MO, USA), digitally photographed with Gel Doc 2000 (Bio-Rad Laboratories, Inc.), and normalized as TIFF images (BioNumerics 6.6 software Applied Math NV, Sint-Martens-Latem, Belgium) using the PFGE global standard *Salmonella* ser. Braenderup H9812. Pattern clustering was performed using the unweighted pair group method, including arithmetic averages (UPGMA) and the Dice correlation coefficient with a position tolerance of 1.5%. PFGE types with no band difference were considered as 100% similar, respectively genetically indistinguishable, according to Tenover et al. [24]. The Simpson’s index of diversity on the combined PFGE cluster analysis (*Asc*I and *Apa*I) was calculated applying the online tool of comparing partitions (http://www.comparingpartitions.info/).

### 2.3. Sequencing and Analysis of Virulence Genes

PCR amplification was performed for the following genes: *actA* (2121 bp)*, hly* (1587 bp) and *inlA* (2400 bp) using specific primers (Table S1). PCR conditions were as follows: 0.2 pmol/µL of each primer, 2 mM MgCl_2_, 1 mM dNTP-Mix, 0.625U Platinum Taq DNA polymerase (Life Technologies, Carlsbad, CA, USA). PCR cycling conditions for *inlA* and *hly* were 95 °C for 2 min, 35 cycles at 94 °C for 1 min, 58 °C for 40 s and 72 °C for 2.5 min, and a final elongation at 72 °C for 5 min; and for *actA* were 95 °C for 2 min, followed by 35 cycles at 94 °C for 1 min, 62 °C for 40 s and 72 °C for 2.5 min, and a final extension at 72 °C for 5 min. Distilled water was included as a negative control in each PCR. PCR products for *hly* and *inlA* were sequenced using the universal sequencing MLST primers. For sequencing the *actA* PCR products we used the forward and reverse primers of the corresponding PCR reaction (Table S2; LGC Genomics).

Sequences were joined to full length sequences using MAFFT [25] and translated into amino acid sequences (http://web.expasy.org/translate/). The InlA and LLO amino acid sequences of the *L. monocytogenes* strains Ro01-15, included in this study, have been analyzed in a recent project [19]. Amino acid sequences were aligned with MAFFT; alignments were visualized using BOXSHADE (http://www.ch.embnet.org/software/BOX_form.html). The phylogenetic analysis and the estimation of the overall mean distance were performed using MUSCLE implemented in MEGA7 [22]. The analysis involved 57 amino acid sequences. After alignment we used the maximum likelihood method based on the Jones–Taylor–Thornton (JTT) matrix-based model for the phylogenetic analysis, including bootstrap analysis, to estimate the reliability (number of bootstrap repetitions 100) [26] and the Poisson model to estimate the overall mean distance. All positions with less than 75% site coverage—meaning fewer than 25% alignment gaps, missing data, and ambiguous bases—were allowed at any position. This resulted in 800 positions for InlA in the final set, 529 for LLO, and 633 for ActA.

### 2.4. Nucleotide Sequence Accession Number

The nucleotide sequences have been deposited in GenBank under the following accession numbers: *inlA* of isolate 1_5 (ST2): MG922914, 6_3 (ST37): MG922915, Ro05 (ST155): MG922916 6_14 (ST155) MG922917 and 6_20 (ST199) MG922918; *hly* of isolate Ro05 (ST155) MG922919 and 1_5 (ST2) MG922920; and *actA* of isolate Ro05 (ST155): MG922921 and 6_20 (ST199) MG922922. For all other sequences, homologous sequences sharing 100% nucleotide identity were found in the NCBI database using BLAST.

## 3. Results and Discussion

### 3.1. Strain Characteristics and Genotype Diversity

In total, we have isolated 57 *L. monocytogenes* isolates from 1474 food products imported from 17 different non-EU countries (Table 1, Table S1). That corresponds to a prevalence of 3.87%. Of these, 28 isolates originated from Asia, and among them, 50% were from Turkey (*n* = 14) and 21% from Russia (*n* = 6), which reflects the high numbers of Austrian and German immigrants originating from Turkey and Russia. Additionally, 18 isolates originated from non-EU countries in Europe, of these, 83% were from the Republic of Moldova (*n* = 15), 11% from Ukraine (*n* = 2), and one strain was from Albania (Table 1). The origin of ten *L. monocytogenes* isolates was South America. Only one *L. monocytogenes* positive food sample derived from Africa (Table 1).

Sixty-three percent of *L. monocytogenes* isolates derived from meat and meat products (36/57), whereas 11 isolates originated from fish (19%), and nine from dairy products (16%, Table 2, Table S1).

In comparison to illegally imported food products, 343 *L. monocytogenes* notifications in food and feed were reported in the same period (2012 to 2015) within the Rapid Alert System for Food and Feed, which is a system for reporting food safety issues within the EU (RASSF portal; https://ec.europa.eu/food/safety/rasff_en). This represents 11.86% of all notifications on pathogenic microorganisms within the EU. The highest *L. monocytogenes* notification rates were identified in fish (*n* = 127), milk and milk products (*n* = 99), and meat products (*n* = 74).

**Figure 1:**
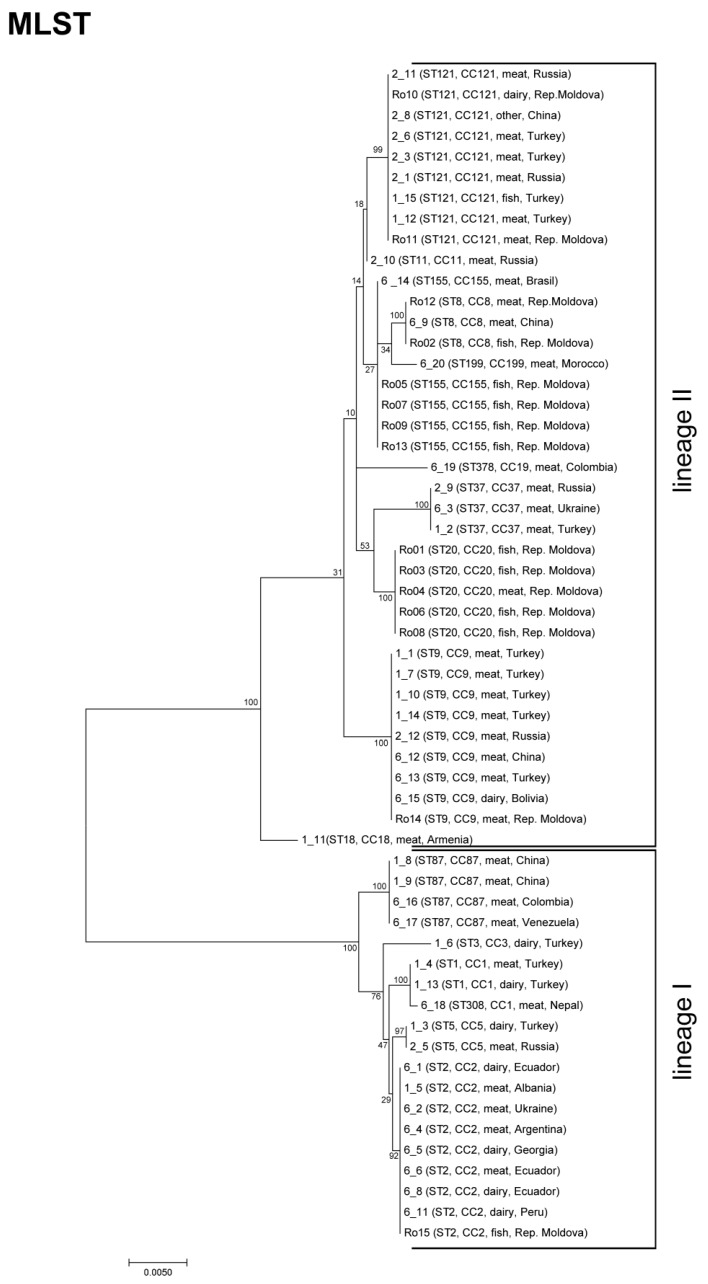
Maximum likelihood phylogenetic tree of *Listeria monocytogenes* strains based on multilocus sequence typing (MLST) loci. The maximum likelihood phylogenetic tree is based on concatenated full-length MLST gene sequences and was calculated with MEGA7 using the Tamura–Nei model. Bootstrap values (100× resampling) are indicated at the respective nodes. The analysis involved 57 nucleotide sequences. All positions containing gaps were eliminated. There was a total of 3288 positions in the final dataset. The bar represents the number of substitutions per site. *L. monocytogenes* sequence types (STs), clonal complexes (CCs), source, and the origin country, are indicated.

Of the 57 isolates, 20 belong to lineage I and 37 strains to lineage II. MLST typing revealed 16 different STs belonging to 15 CCs (Figure 1, Simpson’s diversity index 0.909). The most prevalent STs were ST2, ST9, and ST121 comprising each of nine isolates, followed by ST20 (*n* = 5) and ST155 (*n* = 5). Eight STs comprised only one or two isolates. The predominant genotypes of *L. monocytogenes* isolated from meat and meat products were ST121 and ST9; whereas ST20, ST155, and ST2 were most prevalent in fish and dairy products, respectively (Table 2).

This is in accordance with a recent study from the European Food Safety Authority analyzing the core genome MLST of 1143 *L. monocytogenes* isolates. The 10 predominant CCs (CC121, CC9, CC8, CC1, CC2, CC155, CC87, CC3, CC37, CC5, CC20, and CC18; provided in descending order) were also identified in our study [27].

Due to the diverse origins of the *L. monocytogenes* strains (17 different counties), we initially, would have expected higher genotype diversity. A recent study, including 300 *L. monocytogenes* strains from different sources (human, food, environment, and animals) from 42 countries in five continents showed a high diversity [28]. The 300 isolates represented 111 STs grouped into 17 CCs. The most prevalent CCs were CC2, CC1, and CC3, all belonging to lineage I [28]. Strains of ST2 (CC2) were also highly prevalent in our study; whereas, we detected only three strains of CC1 and one isolate belonging to CC3. Strains of CC1 are known to be overrepresented in clinical isolates [29]. However, our study included only *L. monocytogenes* isolated from food sources. The other two of the three highly abundant STs in our study, mainly ST9 and ST121, are known to be the most abundant STs, at least in Europe, and are overrepresented in food and food-associated environments [27,29]. Moreover, a recent study analyzing the population diversity of *L. monocytogenes* strains of diverse geographical locations showed that ST9 and ST121 have the highest geographical transition rates [30].

Sequence type 155 strains, represented by five isolates in our study, have shown only a moderate prevalence among European and international strains [27,28,29]. Recently, ST155 isolates were involved in human listeriosis cases reported from Denmark [31]. ST20 strains, also represented by five isolates, appear to be rare, and rather environmentally associated, especially to silage and animals [32]. Interestingly, ST199, originating in this study from a meat product in Morocco, was also present in legally sold Moroccan food products (2009–2015) [33].

**Figure 2:**
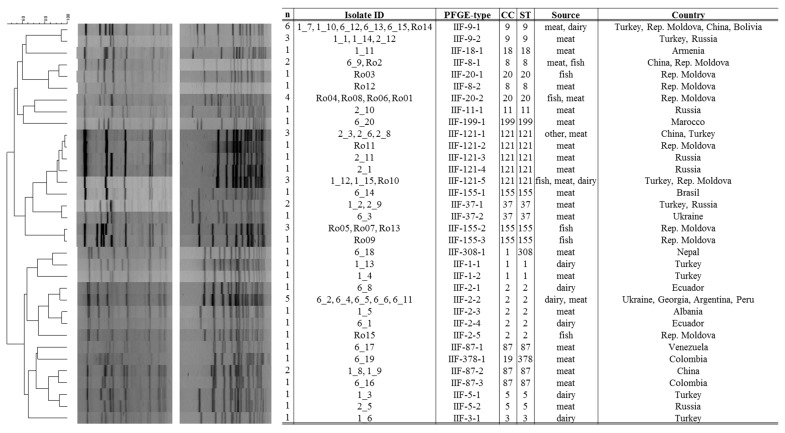
Combined pulsed-field gel electrophoresis (PFGE) cluster analysis of 57 *L. monocytogenes* isolates. Combined PFGE cluster analysis of *L. monocytogenes* isolates from this study (restriction enzymes *Asc*I & *Apa*I). The TIFF images were compared using BioNumerics 6.6 software (Applied Math NV, Sint-Martens-Latem, Belgium), and normalized using the PFGE global standard *Salmonella* ser. Braenderup H9812. Pattern clustering was performed using the unweighted pair group method using arithmetic averages (UPGMA) and the Dice correlation coefficient was applied with a position tolerance of 1.5%.

The PFGE typing of 57 *L. monocytogenes* isolates, with the restriction enzyme *Asc*I and *Apa*I, resulted in 25 and 30 different pulsotypes, respectively. PFGE analyses combining the results obtained with both restriction enzymes identified 34 unique pulsotypes, resulting in a Simpson’s diversity index of 0.971 (Figure 2).

*L. monocytogenes* isolates assigned to PFGE type IIF-9-1, IIF-9-2, IIF-121-1, IIF-121-5, and II-2-2 were 100% similar within their fingerprint profile, and seem to be widely distributed (Figure 2). The latter PFGE profiles were identified as ST9 (IIF-9-1, IIF-9-2), ST121 (IIF-121-1, IIF-121-5; genetic lineage II), and ST2 (II-2-2; genetic lineage I) which are common STs persistent in food processing environments [28,29,34]. Furthermore, indistinguishable isolates were observed in PFGE-profile IIF-8-1, IIF-37-1, and IIF-87-2 (each *n* = 2) corresponding to ST8, ST37, and ST87 (Figure 2). ST8 isolates are also related to persistence, especially in the fish and meat processing chain [34,35]. ST37 representatives are rather rare and were most recently isolated from nature and cheese processing environment [32,36]. ST87 are both human outbreak and food associated and frequently reported from China [37].

Furthermore, indistinguishable PFGE types were also found among isolates from illegally sold fish and meat products at a black market in Romania. In detail, PFGE type IIF-20-2 and IIF-155-2 were identified among 4 and 3 different food products, respectively. Cross-contaminations with the same *Listeria* isolate are highly probable.

In summary, the discriminatory power of PFGE method was superior in comparison with MLST (Simpson’s diversity index of 0.971 versus 0.909), resulting in 34 combined *Asc*I/*Apa*I profiles. Apparently, PFGE typing is a good complementary method to the MLST, and even to genome multilocus sequence typing (cgMLST) where subtypes related to rearrangements in the accessory genome (uptake or loss of prophages, plasmids) can be identified [38].

### 3.2. InlA Variants

**Figure 3:**
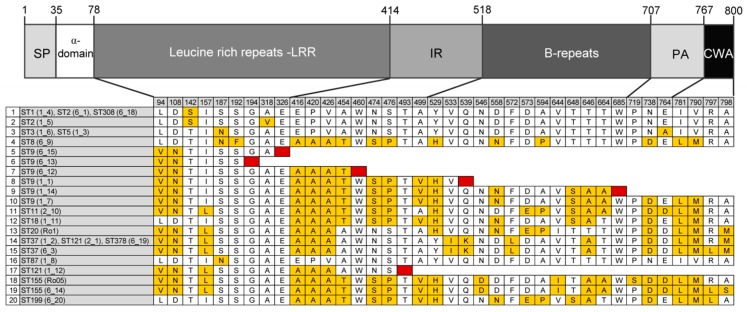
Internalin A variants. Analysis of InlA amino acid sequence of 57 *L. monocytogenes* isolates revealed 20 different variants. InlA functional domains are represented as distinct blocks: signal peptide (SP), leucine rich repeats (LRR), intragenic repeat (IR), B-repeats, pre-anchor domain (PA), and cell wall anchor (CWA). Different variants are presented by one representative strain. Red indicates premature stop codon (PMSC).

We detected a high diversity in the InlA amino acid sequence of the 57 international *L. monocytogenes* isolates with an overall mean distance estimation of 0.013. In total, we detected 20 different variants (Figure 3), and among them, six variants have a truncated InlA due to the presence of a premature stop codon (PMSC).

The truncated form of InlA is secreted, rather than anchored in the cell wall, leading to attenuated virulence and reduced pathogenicity [39,40,41]. To date, 30 different frameshifts and mutations leading to PMSC are known [30]. In total, 14 isolates (24.6%), all belonging to ST9 or ST121, harbor a truncated *inlA* gene (Figure 3). All ST121 isolates, except isolate 2_1, have a PMSC at position 492 (mutation type 6 [42]). ST121 strains, whose genomes are highly conserved, are known to harbor this specific PMSC type in the *inlA* sequence [30,43,44]. Interestingly, isolate 2-1 has a full-length InlA, identical to isolate 6_19 (ST378). ST121 strains carrying a full-length InlA are very rare, and have so far only been reported for a second strain, the human isolate SLCC1589 [43]. ST121 strains, although highly abundant, are known to be underrepresented among human isolates [28,29,45]. A recent study, including in total 1167 ST121 strains, among them, 82 human isolates, reported that ST121 isolates (79%) induced mainly bacteremia, whereas 20% create an infection of the central nervous system [29]. This clearly shows that ST121 strains have the potential to cause listeriosis, despite the presence of a truncated InlA.

The ST9 isolates carry, in contrast to ST121 isolates, different InlA variants, suggesting a higher genetic diversity in this ST group. Three ST9 isolates have a full length InlA. We detected five different InlA PMSCs harbored by six ST9 isolates: at position 194 AA (mutation type 27 [30], strain 6_13), at position 326 AA (mutation type 19 [46], strain 6_15), at position 460 AA (mutation type 8 [47], strain 6_12 and Ro14), at position 539 (mutation type 14 [20], strain 1_1), and at position 685 (mutation type 11 [47], strain 1_14). In a recent study analyzing the genome of 1696 *L. monocytogenes* strains, including 70 ST9 strains, seven different mutations leading to a PMSC in the *inlA* gene have been reported [30].

Further, we detected alterations in all structural motifs of InlA, except the signal peptide and the α-domain (Figure 3). The most conserved region of InlA among the 57 *L. monocytogenes* isolates was the LRR domain (2.08% of all AA positions), which is fundamental for the binding to E-cadherin to the host cell [48]. By contrast, the cell wall anchoring (CWA) domain, at the carboxyl-terminal including the LPXTG motif, which anchors the protein to peptidoglycan of the bacterial cell wall [49], and the inter-repeat (IR) domain, showed a high variation in 12.12% and 6.73% of amino acid positions, respectively. In parallel, Ragon et al. have reported a highly constrained LRR region, and moderately constrained IR and B-repeat regions [20].

**Figure 4:**
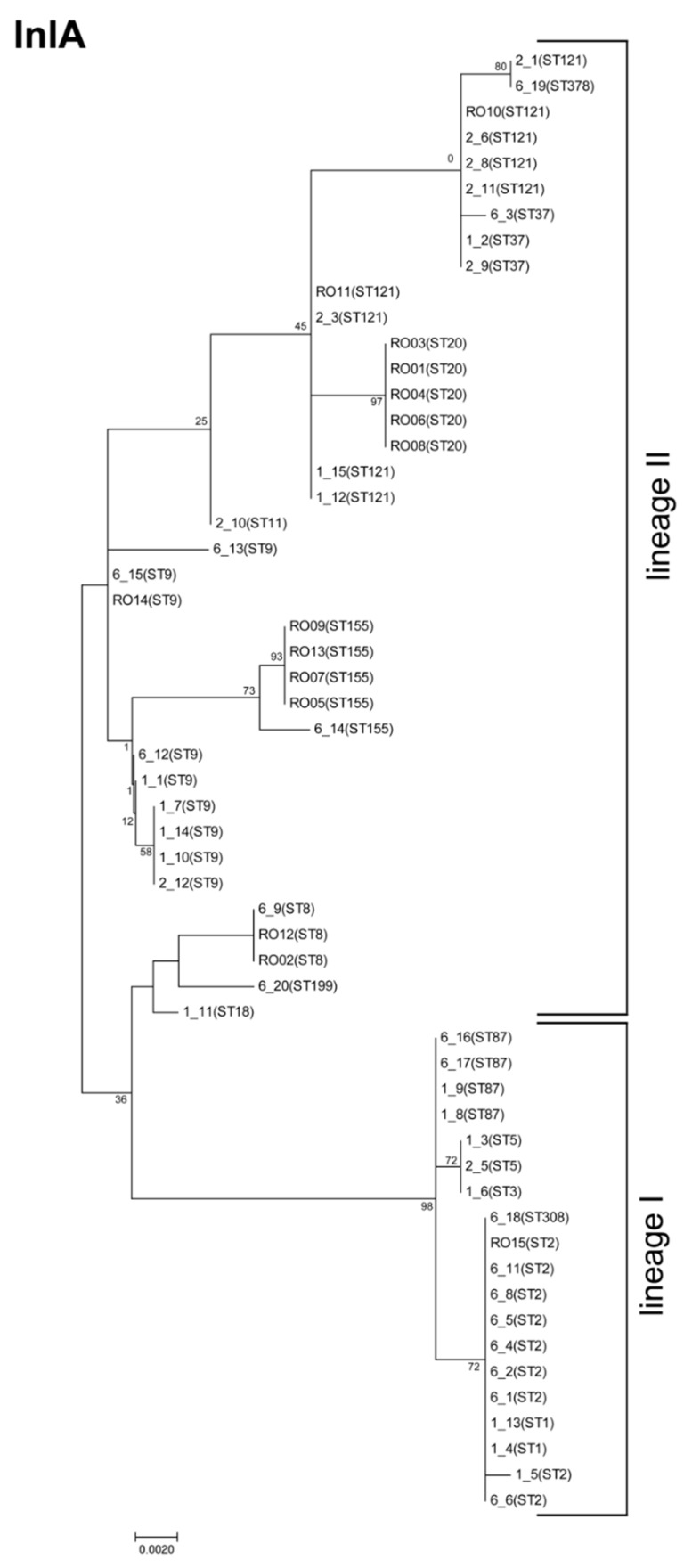
Phylogenetic analysis of internalin A. The molecular phylogenetic analysis by the maximum likelihood method is based on InlA amino acid sequences, and was calculated with MEGA7 using the Jones–Taylor–Thornton (JTT) matrix-based model [22,26]. The tree is drawn to scale, with branch lengths measured by the number of substitutions per site. Bootstrap values (100× resampling) are indicated at the respective nodes. The analysis involved 57 amino acid sequences. All positions with less than 75% site coverage were eliminated, resulting in 800 positions in the final dataset.

The phylogenetic tree, including the InlA amino acid sequences of all 57 *L. monocytogenes* isolates (site coverage ≥75%), reveals that not all isolates of the same ST harbor the same InlA variant, and that isolates of different STs can carry the same InlA variant (Figure 4).

### 3.3. Listeriolysin O Variants

**Figure 5:**
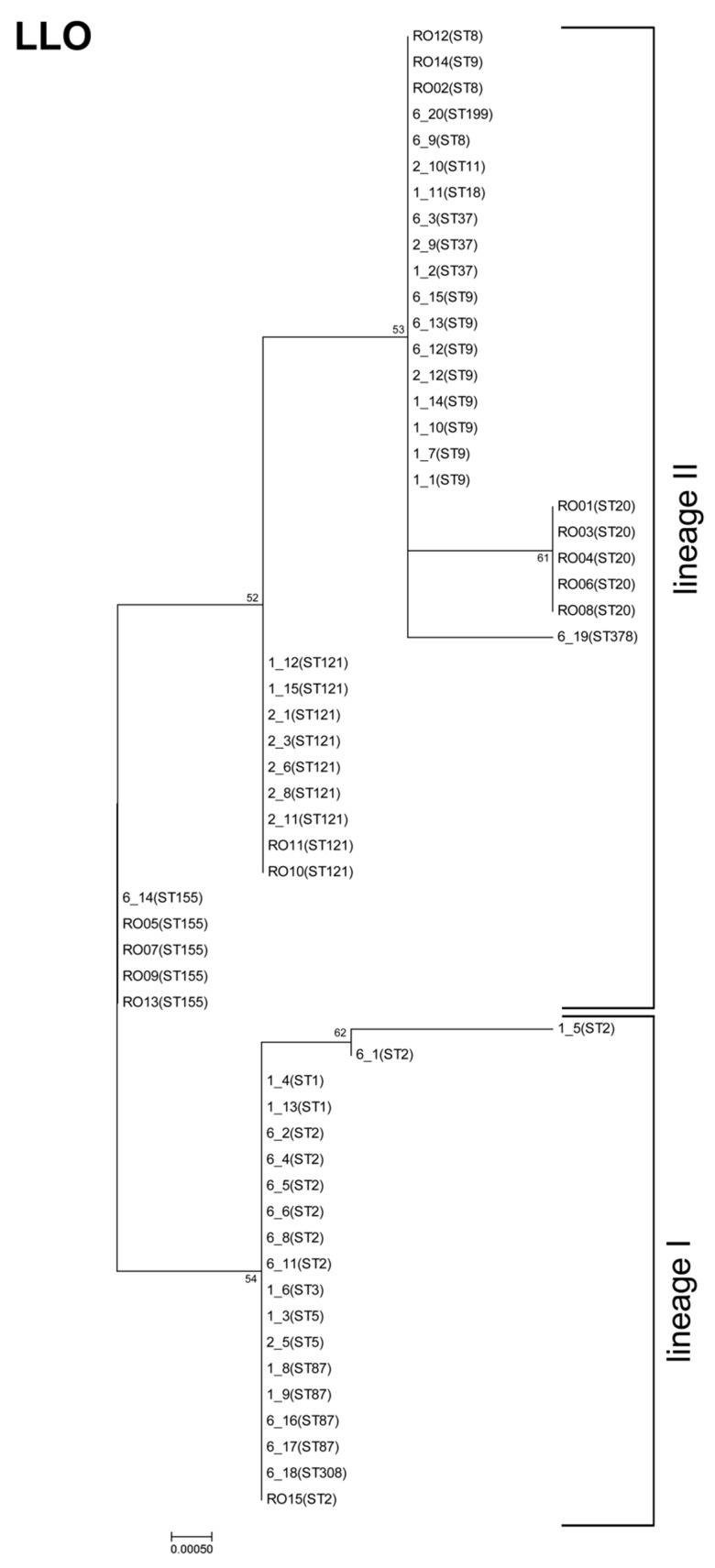
Phylogenetic analysis of listeriolysin O (LLO). The molecular phylogenetic analysis by the maximum likelihood method is based on InlA amino acid sequences, and was calculated with MEGA7 using the JTT matrix-based model [22,26]. The tree is drawn to scale, with branch lengths measured in the number of substitutions per site. Bootstrap values (100× resampling) are indicated at the respective nodes. The analysis involved 57 amino acid sequences. All positions with less than 75% site coverage were eliminated, resulting in 529 positions in the final dataset.

We also sequenced the *hly* gene and analyzed the corresponding amino acid sequence of LLO, a pore-forming toxin of the CDC family. We detected a lower diversity in the LLO amino acid sequence compared to InlA, with an overall mean distance estimation of 0.003 (shown also in the phylogenetic tree, Figure 5).

Listeriolysin O was present in eight different variants. Isolates belonging to the same ST harbored the same LLO sequence (except the two ST2 strains). The phylogenetic tree clearly shows a distinction among the LLOs of isolates from lineage I and II (Figure 5). This is in line with previous studies showing that distinct genetic lineages of *L. monocytogenes* have characteristic ribotypes with the *hly* allelic types [50].

One isolate (1_5, ST2) carries a PMSC in the *hly* gene, resulting in a truncated LLO (293 AA), which lacks the pH regulation domain and the cholesterol-binding motif. PMSCs in the *hly* gene appear to be very rare. So far, only one study that analyzed the genome of 104 strains reported the presence of truncated LLO in one strain, which was also from CC2 (ST553) [29]. However, it remains unclear whether PMSC in the *hly* gene is restricted to strains of CC2 (lineage I).

We observed an alternation in the N-terminus at position 35 (L/S), the region containing the 26 aa PEST motif (including the amino acids P, E, S, and T), which is involved in phagosomal escape of bacteria in infected cells [51,52,53]. Further, we observed alterations in the CDC four-domain structure at positions 119 (N/D) and 185 (V/I); near the pH regulation domain at positions 375 (V/I), 433 (V/I), and 438 (V/I); and at the C-terminus at positions 523 (S/K), 524 (D/V), and 525 (D/V). Various studies have shown that mutations in the pH regulation domain and at the C terminus, which includes the cholesterol binding motif, attenuate the virulence of *L. monocytogenes* [54,55,56,57].

### 3.4. ActA Variants

Among all three of the analyzed virulence factors, ActA showed the second highest variation with an overall mean distance estimation of 0.272 (compared to 0.013 for InlA and 0.003 for LLO). The high overall mean distance estimation factor reflects the presence of internal truncation in ActA, which is harbored by 24.6% of isolates (*n* = 14).

**Figure 6:**
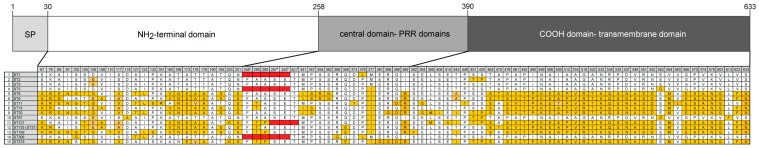
ActA variants. Analysis of ActA amino acid sequence of 57 *L. monocytogenes* isolates revealed 15 different variants. ActA functional domains are represented as distinct blocks. SP: signal peptide, PRR: proline reach repeat; Different variants are presented by isolates of the same ST with two exceptions. ST37 and ST155 isolates harbor the same ActA variant. Red indicates internal truncation. ^a^ start and ^b^ end of the internal truncation.

We detected four ActA variants with an internal truncation: one of which was present in all ST121 isolates (lineage II). Additionally, all ST1, ST5, and ST308 isolates (all lineage I) carry an internal ActA truncation (Figure 6). All these short ActA variants, comprising of 598 amino acids, harbor only three proline rich repeats (PRR) domains compared to four PRR domains (633 amino acids). Internal truncation of ActA appears to be common, and also occurs in strains of other STs [29].

In total, we detected 15 different ST specific variants with one exception: ST37 and ST155 isolates carry the same ActA variant (Figure 6). Variations in the amino acid sequence occurred in all structural domains of ActA, except the signal peptide domain. Variations were present in 10.5% of the amino acids of the N-terminus, which contain the actin-binding domain and other regions required for the initiation of actin polymerization [58], in 11.4% of amino acids of the central region containing either three or four PRR separated by long-repeat regions [59], and in 16.5% of the amino acids of the C-terminus, containing the transmembrane domain responsible for anchoring ActA to the bacterial surface [58]. The central domain of ActA is not required for actin-based motility, but influences the speed of movement [60,61]. Studies examining the effects of different ActA variants on the virulence potential of *L. monocytogenes* are rare and have produced inconsistent results [18,62].

**Figure 7:**
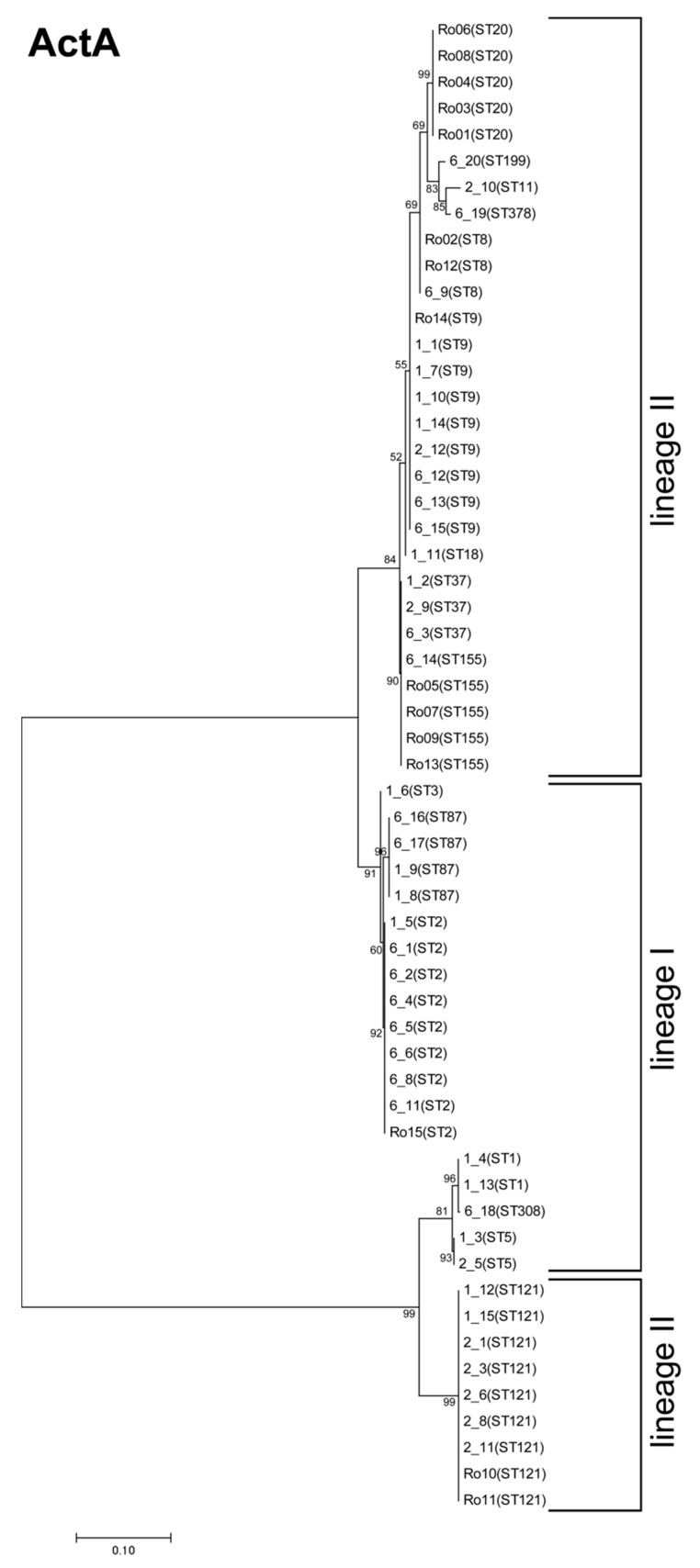
Phylogenetic analysis of ActA. Molecular phylogenetic analysis, based on the maximum likelihood method using ActA sequence, was calculated with MEGA7 using the JTT matrix-based model [22,26]. The tree is drawn to scale, with branch lengths measured by the number of substitutions per site. Bootstrap values (100× resampling) are indicated at the respective nodes. The analysis involved 57 amino acid sequences. All positions with less than 75% site coverage were eliminated resulting in 633 positions in the final dataset.

The phylogenetic tree of ActA shows a clear distinction between isolates with and without an internal truncation (Figure 7). We observed an additional distinction between isolates of lineage I and II in these two clusters.

## 4. Conclusions

In this study, we characterized 57 *L. monocytogenes* isolates, which derived from 1474 illegally imported food products into the EU. The presence of *L. monocytogenes* in these types of samples uncovers new routes of pathogen transmission, which can endanger human health.

Although the *L. monocytogenes* strains originated from 17 different countries from four continents, we revealed moderate genotype diversity based on MLST and PFGE typing (16 STs belonging to 15 CCs and 34 unique pulsotypes). The most prevalent STs (ST121, ST9, ST2) of these international isolates are known to be among the most abundant STs, at least in Europe, underlining the global spread of certain STs. Two STs (ST20 and ST155), reported to be only rarely present, were highly abundant among food from the Romanian black market, showing a high regional prevalence of these STs.

The further characterization of virulence markers (InlA, LLO, and ActA) was important for an initial risk categorization. The highest diversity in virulence markers was, overall, observed within InlA (20 different InlA variants), and 14 strains (ST9 and ST121) harbored PMSC, indicating an attenuated virulence potential. An internally truncated ActA (*n* = 4) was identified among both genetic lineage I (ST1, ST5, and ST308) and II (ST121) strains. Interestingly, ST37 and ST155 carried the same ActA variant. LLO clearly separated genetic lineage I and II strains, but resulted in the lowest diversity (8 genetic variants).

Further studies, including a higher number of international *L. monocytogenes* isolates and using whole genome sequencing, will be necessary to study the population diversity of *L. monocytogenes* world-wide.

## Figures and Tables

**Table 1:** Origin of *Listeria monocytogenes* isolates assigned to sequence types (STs).

Continent	Country	STs
Africa (*n* = 1)	Morocco (*n* = 1)	199 (*n* = 1)
Asia (*n* = 28)	Armenia (*n* = 1)	18 (*n* = 1)
	China (*n* = 5)	8, 9, 121 (*n* = 1); 87 (*n* = 2)
	Georgia (*n* = 1)	2 (*n* = 1)
	Nepal (*n* = 1)	308 (*n* = 1)
	Russia (*n* = 6)	5, 9, 11, 37 (*n* = 1); 121 (*n* = 2)
	Turkey (*n* = 14)	3, 5, 37 (*n* = 1); 1 (*n* = 2); 121 (*n* = 4); 9 (*n* = 5)
Europe (*n* = 18)	Albania (*n* = 1)	2 (*n* = 1)
	Republic of Moldova (*n* = 15)	2, 9 (*n* = 1); 8, 21 (*n* = 2); 155 (*n* = 4); 20 (*n* = 5)
	Ukraine (*n* = 2)	2, 37 (*n* = 1)
South America (*n* = 10)	Argentina (*n* = 1)	2 (*n* = 1)
	Bolivia (*n* = 1)	9 (*n* = 1)
	Brazil (*n* = 1)	155 (*n* = 1)
	Colombia (*n* = 2)	87, 378 (*n* = 1)
	Ecuador (*n* = 3)	2 (*n* = 3)
	Peru (*n* = 1)	2 (*n* = 1)
	Venezuela (*n* = 1)	87 (*n* = 1)

**Table 2:** *L. monocytogenes* ST distribution among different food categories.

Food Source	STs
Dairy (*n* = 9)	1, 3, 5, 9, 121 (*n* = 1)
	2 (*n* = 4)
Fish (*n* = 11)	2, 8, 121 (*n* = 1)
	20, 155 (*n* = 4)
Meat (*n* = 36)	1, 5, 11, 18, 20, 155, 199, 308, 378 (*n* = 1)
	8 (*n* = 2)
	37 (*n* = 3)
	2, 87 (*n* = 4)
	121 (*n* = 6)
	9 (*n* = 8)
Other (*n* = 1)	121 (*n* = 1)

## Supplementary Materials

The following are available online at http://www.mdpi.com/2073-4425/9/9/428/s1. Table S1: Strain characteristics, Table S2: Primers used in this study.
