# Supplementary material for: Listeria monocytogenes Isolated from Illegally Imported Food Products into the European Union Harbor Different Virulence Factor Variants
Source: Genes (Basel). 2018 Aug 23;9(9):428. doi: 10.3390/genes9090428 (PMC6162745; doi:10.3390/genes9090428)
Supplement: Supplementary file 1 [file genes-09-00428-s001.zip › Table S2.docx]

**Table S2**: Primers used in this study

| **Target gene** | **Primers** | **Product size (bp)** |
| --- | --- | --- |
| *actA* | Fwd^1^: GTATTAGCGTATCACGACGA  Rev1^1^: CAAGCACATACCTAGAACCA | 2,121 |
|  | Fwd: GTATTAGCGTATCACGACGA  Rev2^1^: CCCGCATTTCTTGAGTGTTT | 1,352 |
| *inlA* | Fwd1: GTTTTCCCAGTCACGACGTTGTACATGATTTTTCGGATGCAGGAG Rev: TTGTGAGCGGATAACAATTTCACTATCCTCTCCTTGATTCTAG | 2,400 |
|  | Fwd2: GTTTTCCCAGTCACGACGTTGTAAGTCTTATCGCTACTAACAACC  Rev: TTGTGAGCGGATAACAATTTCACTATCCTCTCCTTGATTCTAG | 1,791 |
| *hly* | Fwd: GTTTTCCCAGTCACGACGTTGTATGCGTTTCATCTTTAGAAGC  Rev: TTGTGAGCGGATAACAATTTCAAGCCTGTTTCTACATTCTTCA | 1,587 |
| MLST primers^2^ | Fwd: GTTTTCCCAGTCACGACGTTGTA  Rev: TTGTGAGCGGATAACAATTTC |  |

^1^Primers used for sequencing of the *actA* gene, ^2^universal MLST primers used for sequencing
